# Audit on Tissue Box Availability in Outpatient Psychiatric Consultation Rooms

**DOI:** 10.1192/bjo.2026.11834

**Published:** 2026-06-30

**Authors:** Sasha Saba, Animesh Tripathi

**Affiliations:** Hertfordshire Partnership NHS Foundation Trust, Hatfield, United Kingdom

## Abstract

**Aims:**

Psychiatric consultations often involve emotionally challenging discussions that can be difficult for patients. In the event where tissue boxes are not available on hand, this can lead to interruptions and negatively impact patient engagement. The immediate availability of tissue boxes in clinic rooms is a simple yet powerful tool to achieve compassionate care and build rapport. As far as we are aware, there is no policy regarding this practice within our trust which is highly variable between sites. The immediate aim of the audit is to assess the availability of tissue boxes in psychiatric consultation rooms in a predefined week across three different community bases and to compare results to a consensually agreed set gold standard of 100% availability. The broader aim was to then evidence improvement in practice following a therapeutic intervention with a view to sustain good practice moving forward

**Methods:**

A cross-sectional evaluation was conducted across three outpatient psychiatric settings during a predefined one-week period. Each clinic room was visited once, and the presence or absence of a tissue box was recorded. The evaluation was conducted against a set gold standard of 100% tissue box availability in all rooms. Results were analysed and as an intervention these were disseminated to relevant site managers with a recommendation to ensure all rooms are stocked with tissues. A re-audit was then conducted to assess improvement and compliance with our set gold standard.

**Results:**

A total of 33 clinic rooms across the different locations were assessed. Initially, it was found that 10/12 clinic rooms had a positive result (presence of tissue box in the room) in location (1) which equals an estimate of 83.4%. This result remained the same post intervention. For location (2), 8/9 clinic rooms (88.9%) had a positive result, and this also remained the same post intervention. It was found that location (3) had the lowest rate of positive results with only 5/12 rooms prior intervention (50%). This was reduced to 41% post intervention.

**Conclusion:**

Whilst there are baseline differences between sites, the overall results evidenced good practice. The marginal improvement would suggest the need for a more robust intervention to produce consistent change across sites. The presence of tissue boxes in clinic rooms is likely to have a significant impact on positive patient experience. Therefore, moving forward, we hope to assess this practice across all HPFT and incorporate a policy guidance to ensure compliance.

